# Supplementary material for: Deletion of wfs1 Impairs Oligodendrocyte Precursor Cells Dorsal Distribution and Myelination Through the wfs1-hmgcs1 Axis in Zebrafish
Source: Biology (Basel). 2026 Aug 16;15(16):1402. doi: 10.3390/biology15161402 (PMC13510151; doi:10.3390/biology15161402)
Supplement: Supplementary file 1 [file biology-15-01402-s001.zip › File S1-Transcriptome sequencing data and differentially expressed genes (DEGs) in wfs1-- zebrafish at 5 dpf/1.GO/wfs1KOvsWT/ALL/wfs1KOvsWT.all.GObp_DAG.pdf]

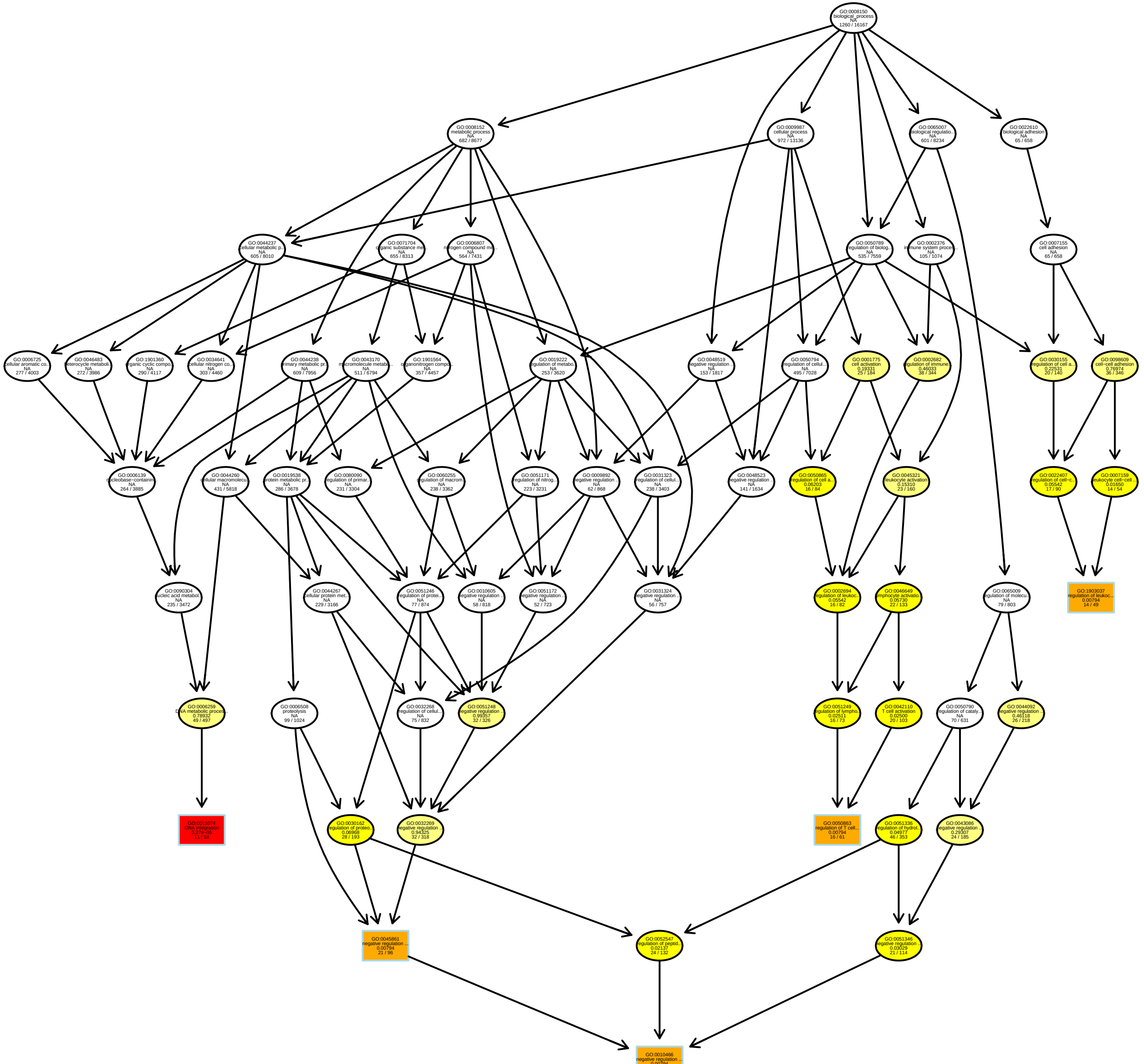

GO:0008150 biological_process NA 1260 / 16167
GO:0008152 metabolic process NA 682 / 8677
GO:0009987 cellular process NA 972 / 13136
GO:0065007 biological regulatio... NA 601 / 8234
GO:0022610 biological adhesion NA 65 / 658
GO:0044237 cellular metabolic p... NA 605 / 8010
GO:0071704 organic substance me... NA 655 / 8313
GO:0006807 nitrogen compound me... NA 564 / 7431
GO:0050789 regulation of biolog... NA 535 / 7559
GO:0002376 immune system proces... NA 105 / 1074
GO:0007155 cell adhesion NA 65 / 658
GO:0006725 cellular aromatic co... NA 277 / 4003
GO:0046483 heterocycle metaboli... NA 272 / 3986
GO:1901360 organic cyclic compo... NA 290 / 4117
GO:0034641 cellular nitrogen co... NA 303 / 4460
GO:0044238 primary metabolic pr... NA 609 / 7956
GO:0043170 macromolecule metabo... NA 511 / 6794
GO:1901564 organonitrogen compo... NA 357 / 4457
GO:0019222 regulation of metabo... NA 253 / 3620
GO:0048519 negative regulation ... NA 153 / 1817
GO:0050794 regulation of cellul... NA 495 / 7028
GO:0001775 cell activation 0.19331 25 / 184
GO:0002682 regulation of immune... 0.46033 38 / 344
GO:0030155 regulation of cell a... 0.22531 20 / 140
GO:0098609 cell−cell adhesion 0.76974 36 / 346
GO:0006139 nucleobase−containin... NA 264 / 3885
GO:0044260 cellular macromolecu... NA 431 / 5818
GO:0019538 protein metabolic pr... NA 286 / 3678
GO:0080090 regulation of primar... NA 231 / 3304
GO:0060255 regulation of macrom... NA 238 / 3362
GO:0051171 regulation of nitrog... NA 223 / 3231
GO:0009892 negative regulation ... NA 62 / 868
GO:0031323 regulation of cellul... NA 238 / 3403
GO:0048523 negative regulation ... NA 141 / 1634
GO:0050865 regulation of cell a... 0.06203 16 / 84
GO:0045321 leukocyte activation 0.15310 23 / 160
GO:0022407 regulation of cell−c... 0.05542 17 / 90
GO:0007159 leukocyte cell−cell ... 0.01650 14 / 54
GO:0090304 nucleic acid metabol... NA 235 / 3472
GO:0044267 cellular protein met... NA 229 / 3166
GO:0051246 regulation of protei... NA 77 / 874
GO:0010605 negative regulation ... NA 58 / 818
GO:0051172 negative regulation ... NA 52 / 723
GO:0031324 negative regulation ... NA 56 / 757
GO:0002694 regulation of leukoc... 0.05542 16 / 82
GO:0046649 lymphocyte activatio... 0.05730 22 / 133
GO:0065009 regulation of molecu... NA 79 / 803
GO:1903037 regulation of leukoc... 0.00794 14 / 49
GO:0006259 DNA metabolic proces... 0.78932 49 / 497
GO:0006508 proteolysis NA 99 / 1024
GO:0032268 regulation of cellul... NA 75 / 832
GO:0051248 negative regulation ... 0.99357 32 / 326
GO:0051249 regulation of lympho... 0.02511 16 / 73
GO:0042110 T cell activation 0.02500 20 / 103
GO:0050790 regulation of cataly... NA 70 / 631
GO:0044092 negative regulation ... 0.46118 26 / 218
GO:0015074 DNA integration 3.2e−05 11 / 18
GO:0030162 regulation of proteo... 0.06968 28 / 193
GO:0032269 negative regulation ... 0.94325 32 / 318
GO:0050863 regulation of T cell... 0.00794 16 / 61
GO:0051336 regulation of hydrol... 0.04977 46 / 353
GO:0043086 negative regulation ... 0.29307 24 / 185
GO:0045861 negative regulation ... 0.00794 21 / 96
GO:0052547 regulation of peptid... 0.02137 24 / 132
GO:0051346 negative regulation ... 0.03029 21 / 114
GO:0010466 negative regulation ... 0.00794 20 / 86
